# Anhedonic behavior in cryptochrome 2-deficient mice is paralleled by altered diurnal patterns of amygdala gene expression

**DOI:** 10.1007/s00726-015-1968-3

**Published:** 2015-03-28

**Authors:** Giorgia Savalli, Weifei Diao, Stefanie Berger, Marianne Ronovsky, Timo Partonen, Daniela D. Pollak

**Affiliations:** 1 Department of Neurophysiology and Neuropharmacology, Center for Physiology and Pharmacology, Medical University of Vienna, Schwarzspanierstrasse, 17, 1090 Vienna, Austria; 2 Department of Health, National Institute for Health and Welfare, Helsinki, Finland

**Keywords:** Cry2, Amygdala, Depression, Anhedonia, Clock gene, VEGF

## Abstract

Mood disorders are frequently paralleled by disturbances in circadian rhythm-related physiological and behavioral states and genetic variants of clock genes have been associated with depression. *Cryptochrome 2* (*Cry2*) is one of the core components of the molecular circadian machinery which has been linked to depression, both, in patients suffering from the disease and animal models of the disorder. Despite this circumstantial evidence, a direct causal relationship between *Cry2* expression and depression has not been established. Here, a genetic mouse model of *Cry2* deficiency (*Cry2* ^−/−^ mice) was employed to test the direct relevance of *Cry2* for depression-like behavior. Augmented anhedonic behavior in the sucrose preference test, without alterations in behavioral despair, was observed in *Cry2* ^−/−^ mice. The novelty suppressed feeding paradigm revealed reduced hyponeophagia in *Cry2* ^−/−^ mice compared to wild-type littermates. Given the importance of the amygdala in the regulation of emotion and their relevance for the pathophysiology of depression, potential alterations in diurnal patterns of basolateral amygdala gene expression in *Cry2* ^−/−^ mice were investigated focusing on core clock genes and neurotrophic factor systems implicated in the pathophysiology of depression. Differential expression of the clock gene *Bhlhe40* and the neurotrophic factor *Vegfb* were found in the beginning of the active (dark) phase in *Cry2* ^−/−^ compared to wild-type animals. Furthermore, amygdala tissue of *Cry2* ^−/−^ mice contained lower levels of *Bdnf*-*III*. Collectively, these results indicate that *Cry2* exerts a critical role in the control of depression-related emotional states and modulates the chronobiological gene expression profile in the mouse amygdala.

**Electronic supplementary material:**

The online version of this article (doi:10.1007/s00726-015-1968-3) contains supplementary material, which is available to authorized users.

## Introduction

*Cryptochrome 2* (*Cry2*) is one of the key components of the mammalian intrinsic clock (Griffin et al. 1999; van der Horst et al. 1999; Vitaterna et al. 1999). The genetic core of circadian clocks is constituted of about 20 clock genes, whose expression is regulated by transcriptional and translational feedback loops (Albrecht 2012). In this system, Cry2, together with the clock proteins Cry1, Per1 and Per2, forms a heterodimeric repressor complex which attenuates the Clock/Npas2-Arntl(Bmal1)/Arntl2(Bmal2) activator complex (Gekakis et al. 1998; Kume et al. 1999). In contrast to most core elements of the molecular time keeping system (including Clock, Npas2, Arntl, Arntl2 and the Per proteins), the cryproteins have no Per-ARNT-Sim (PAS) domain (Hsu et al. 1996). However, they have been identified to be required for the maintenance of circadian rhythms. Albeit the fact that absence of either gene alone modulates the period length of the free-running circadian rhythm in opposing directions, the concomitant deletion of both proteins results in a total abolition of free-running rhythmicity (van der Horst et al. 1999).

Disturbances in circadian rhythm-related physiological and behavioral states are frequently observed in mood disorders (Carpenter and Bunney 1971; Branchey et al. 1982; von Zerssen et al. 1987; Souetre et al. 1988, 1989; Benca et al. 1992). Interestingly, although the suprachiasmatic nucleus (SCN) of the hypothalamus constitutes the master organizer of the body’s molecular circadian rhythm, clock genes are also expressed in other regions of the brain, including those relevant to the pathophysiology of depression, such as the amygdala and the hippocampus (Lamont et al. 2005; Jilg et al. 2010; Li et al. 2013; Savalli et al. 2014). *Cry2* is highly expressed in the SCN, albeit information on its rhythmic oscillation is controversial (Kume et al. 1999; Mendoza et al. 2005). A diurnal pattern of its expression has been also observed in the amygdala, where it may additionally exert non-circadian functions. Indeed, the observed rhythmic oscillation of *Cry2* in the amygdala is disrupted in an animal model of a mood disorder characterized by increased anhedonic behavior following chronic mild stress (Savalli et al. 2014). Furthermore, mice displaying higher trait-anxiety behavior (HAB) and comorbid depression-like behavior (Sah et al. 2012) also express lower levels of *Cry2* in the hippocampus as compared to normal anxiety/depression-like behavior (NAB) mice (Griesauer et al. 2014). These observations suggest that altered circadian rhythms and depression-related behavior may be linked at the genetic level. Specifically, they point towards a key role for *Cry2*, supported by genetic studies in the human population highlighting the association of *Cry2* with mood disorders and their depressive episodes (Lavebratt et al. 2010; Sjoholm et al. 2010; Kovanen et al. 2013).

Despite this circumstantial evidence for a role of *Cry2* in the pathophysiology of mood disorders, a causal link between altered *Cry2* expression and depression-like behavior has not been established so far. The present study aimed at investigating the direct consequences of *Cry2*-deficiency on depression-like behavior in the mouse and to determine its molecular signature in the basolateral amygdala. Specifically, we investigated whether the lack of Cry2 causes changes diurnal pattern in clock gene expression in amygdala. Additionally, given the critical importance of growth factor support and deficiencies thereof in the pathophysiology of depression, we further aimed to investigate whether *Cry2*-deficiency affects expression of the vascular endothelial growth factor (VEGF) and of the brain derived neurotrophic factor (BDNF), which have been both associated to depressive disorders (as reviewed in Yu and Chen 2011; Clark-Raymond and Halaris 2013).

## Materials and methods

Male adult *Cry2* knock-out (*Cry2* ^−*/*−^) and wild-type littermates (*Cry2* ^+*/*+^) maintained at a C57BL/6J-background (B6.129P2-*Cry2* ^*tm1Asn*^/J) were used (Jackson Laboratory, MA, USA). This strain was created by replacement of the flavine adenine dinucleotide (FAD) binding domain by a neomycin resistance cassette (Thresher et al. 1998; http://jaxmice.jax.org/). Mice were 9–10 weeks old at the onset of experiments. Animals were single-housed in a sound-attenuated room with constant temperature of ≈21 °C under a 12:12-h light/dark cycle (lights on at 6:00 a.m.). The light intensity at the level of the animal cages was ≈200 lux. Food and water were freely available throughout the experiment, unless otherwise specified. All experiments were designed to reduce animal suffering and keep the number of animals used at the minimum level. Animal experiments described in this study were approved by the national ethical committee on animal care and use (BMWF-66.009/0302-II/3b/2013; Bundesministerium für Wissenschaft und Forschung) and carried out according to international laws and policies.

### Behavioral experiments

All behavioral tests were performed during the light cycle. Mice were habituated to the experimental room 30 min before each test. The same cohort of animals (wild-type, *Cry2* ^+/+^ *n* = 12; *Cry2* ^−/−^ *n* = 12) was used in all tests. The order in which tests were performed followed recommendations ranking the tests from least to more stressful (McIlwain et al. 2001) as follows: sucrose preference test, open field, Rota Rod, light/dark box, elevated plus maze, forced swim, tail suspension, novelty-suppressed feeding test. Prior to the initiation of behavioral testing, a gross evaluation of basic neurological function was carried out following an established protocol (Irwin 1968). In agreement with an earlier report (Thresher et al. 1998), no detectable differences in basic neurological performance were observed between *Cry2* ^+/+^ and *Cry2* ^−/−^ mice. A break of at least 24 h was applied between individual tests. All experiments were made by an investigator blind to the genotype of each animal.

#### Sucrose preference test (SPT)

The SPT was carried out essentially as previously described (Khan et al. 2014). Briefly, prior to testing, mice were habituated to drink a 2 %-sucrose solution, during 4 days of training. On day 1 of the training, mice were deprived of food and water for 18 h. On day 2 and 3, mice were given a 2 %-sucrose solution and food was restored. On day 4, the sucrose solution was replaced with tap water for 6 h. Animals were then deprived of food and water and tested for sucrose preference 18 h later. During the test, subjects were given a free choice between two bottles, one with the sucrose solution and the other with water. Mice were tested over 3 h, starting at 9:00 a.m. To prevent possible effects of side preference in drinking behavior, the position of the bottles (right or left of the feeding compartment) was alternated between animals. Total liquid consumption was measured by weighing the bottles before and after the SPT. Sucrose preference was calculated according to the formula: percentage of preference = (sucrose intake/total intake) × 100.

#### Novelty-suppressed feeding test (NSF)

The NSF test measures the latency(s) of the subject to the first feeding event in a novel environment. It was performed essentially as described by Mineur et al. (2007) with minor modifications. The testing apparatus consisted of a clear Plexiglas arena (33 × 47 × 17 cm), brightly lit (800 lux). At the beginning of the experiment, each subject was placed in the corner of the novel arena with a food pellet positioned in the center and the latency to the first bite of food was recorded (maximum time 600 s). After the test food consumption in the home cage was evaluated during 5 min.

#### Forced swim test (FST)

The FST was conducted as previously described (Monje et al. 2011a). Behavior patterns were tracked by VIDEOTRACK (PORSOLT) software provided (Viewpoint, Champagne au mont d’Or, France) during a 6-min session. The last 4 min of the test were used to assess the percentage of time spent immobile.

#### Tail suspension test (TST)

The TST was conducted as previously described (Monje et al. 2011a), with minor modifications. Mice were securely fastened by the distal end of the tail to a metallic hook in a tail suspension system (MedAssociates, St Albans, VT, USA). The presence or absence of immobility, defined as the absence of limb movement, was tracked over a 6-min session by a computational tracking system (Activity Monitor, MedAssociates, St Albans, VT, USA). Percentage of time spent immobile over the total time was assessed.

#### Open field (OF)

The OF followed a protocol previously described (Monje et al. 2011a). Briefly, locomotor activity was monitored by a computational tracking system (Activity Monitor, MedAssociates, St Albans, VT, USA). The total distance covered during the 30 min testing time and the time spent in the center zone were evaluated.

#### Rota Rod test

The Rota Rod test followed a published procedure (Khan et al. 2014). Each mouse was placed separately on one lane of the rotating drum of an automated Rota Rod device (USB Rota Rod ‘SOF-ENV-57X’, MedAssociates, St Albans, VT, USA). The latency to fall off the drum was calculated as the mean value of three consecutive test sessions.

#### Elevated plus maze (EPM)

The EPM was conducted as previously described elsewhere (Shumyatsky et al. 2002). Briefly, the apparatus consisted of a center platform with adjacent two open and two closed arms. Subjects were placed in the center and their behavior was recorded for 5 min by a computational tracking system (Viewpoint, Champagne au mont d’Or, France). Percentage of time spent in open arms and percentage of number of open arm entries were assessed.

#### Light/dark box test (LD)

The LD followed a published protocol with minor modifications (Leach et al. 2013). The testing apparatus consisted of a dark and a lit compartment, connected by an opening between the two (MedAssociates, St Albans, VT, USA). Animals were allowed to freely move between the two compartments, and the percentage of time spent in and the number of entries into the lit compartment were measured by a computational tracking system (Activity Monitor, MedAssociates, St Albans, VT, USA) during a 10-min session.

### Tissue dissection

Mice of both genotypes were randomly divided into two groups and killed by neck dislocation at Zeitgeber Time (ZT) 0.5 (30 min after lights on), or ZT 12.5 (30 min after lights off). Brains were rapidly removed and immediately frozen at −80 °C. Basolateral amygdala samples were collected using a micro-punch procedure (Lamprecht et al. 2002). Briefly, three adjacent brain coronal sections of 300 μm, from the rostral (Bregma −0.94 mm), medial (Bregma −1.24 mm) and caudal (Bregma −1.54 mm) amygdala (Paxinos and Franklin 2001) were collected. Three bilateral samples of basolateral amygdala were extracted with a blunted 0.69 mm inner diameter sample cannula (Fine Science Tools, Heidelberg, Germany), put into 700 μl Qiazol lysis buffer, vortexed and kept at −80 °C until used for further analysis.

## RNA isolation and quantitative real-time PCR (qRT-PCR)

Total RNA was isolated using a commercially available system [miRNA Micro Kit (Quiagen, CA, USA)], amplified using T-Script^®^ reverse transcriptase (QuantiTect Whole Transcriptome, Quiagen, CA, USA) and quantified by the Quant-iT PicoGreen dsDNA Reagent (Invitrogen Corporation, Carlsbad, CA, USA). The reaction for the subsequent qRT-PCR consisted of 2 μl cDNA sample (out of 25 ng/μl dilution of amplified cDNA reaction) and 5.2 μl RNase-free water, together with 7.8 μl primer-specific master mix containing 7.5 μl Power SYBR^®^ Green PCR Master Mix (Applied Biosystems, CA, USA), 0.15 μl forward primer (20 μM) and 0.15 μl reverse primer (20 μM). The thermal cycling profiles were 50 °C for 2 min, 95 °C for 10 min, 40 cycles at 95 °C for 15 s and 60 °C or 62 °C for 1 min. Transcription levels of target genes were assayed in duplicates and normalized against the amount of β-actin mRNA [delta cycle threshold (dCT)]. β-actin was selected as reference gene as its expression was shown to be stable in mouse amygdala even after emotionally challenging conditions (Stork et al. 2001). Data were plotted using the formula: 2^−∆dCT^ (ddCT), where ddCT is the difference between the mean dCT of each group and the mean dCT of the control group at ZT 0.5. Primers used for amplification of *Vegfa*, *Vegfb*, *Vegfc*, *Vegfd*, *Vegfr*-*1*, *Vegfr*-*2*, *Vegfr*-*3* were described previously (Catteau et al. 2011). Primers used for amplification of *Bdnf*-*I*, *Bdnf*-*II*, *Bdnf*-*III*, *Bdnf*-*IV*, *Bdnf*-*V*, *Bdnf* (total) were described previously (Tsankova et al. 2006). Primer sequences for all clock genes analyzed [*Clock*, *Bmal1*, *Cry1*, *Cry2*, *CycloB*, *Dbp*, *E4bp4*, *Id2*, *Npas2*, *Per1*, *Per2*, *Per3*, *Nr1d1* (*Rev*-*erbα*), *Nr1d2* (*Rev*-*erbβ*), *Bhlhe40* (*Dec1*), *Bhlhe41* (*Dec2*), *ROR*-*α*, *ROR*-*β*, *ROR*-*γ*, *NeuroD1*] are listed in Supplementary Material.

### Statistical analysis

Statistical analysis of behavioral experiments was performed using unpaired two-tailed Student’s *t* test. Comparisons of gene expression were based on two-way analysis of variance (ANOVA) (genotype × time point). Post-hoc pairwise comparisons with Bonferroni correction were carried out where significant main effects (*p* < 0.05) had been revealed by the preceding ANOVA. In each instance, significant outlier calculations were performed based upon the extreme studentized deviate methods using the Grubbs’ test. All data were analyzed using SPSS (IBM, SPSS 18.0) statistical software with the alpha level set at 0.05 at all instances.

## Results

### Behavioral analysis of *Cry2*^+*/*+^ and *Cry2*^−*/*−^

#### Cry2^−/−^ mice exhibit lower sucrose preference

**Fig. 1:**
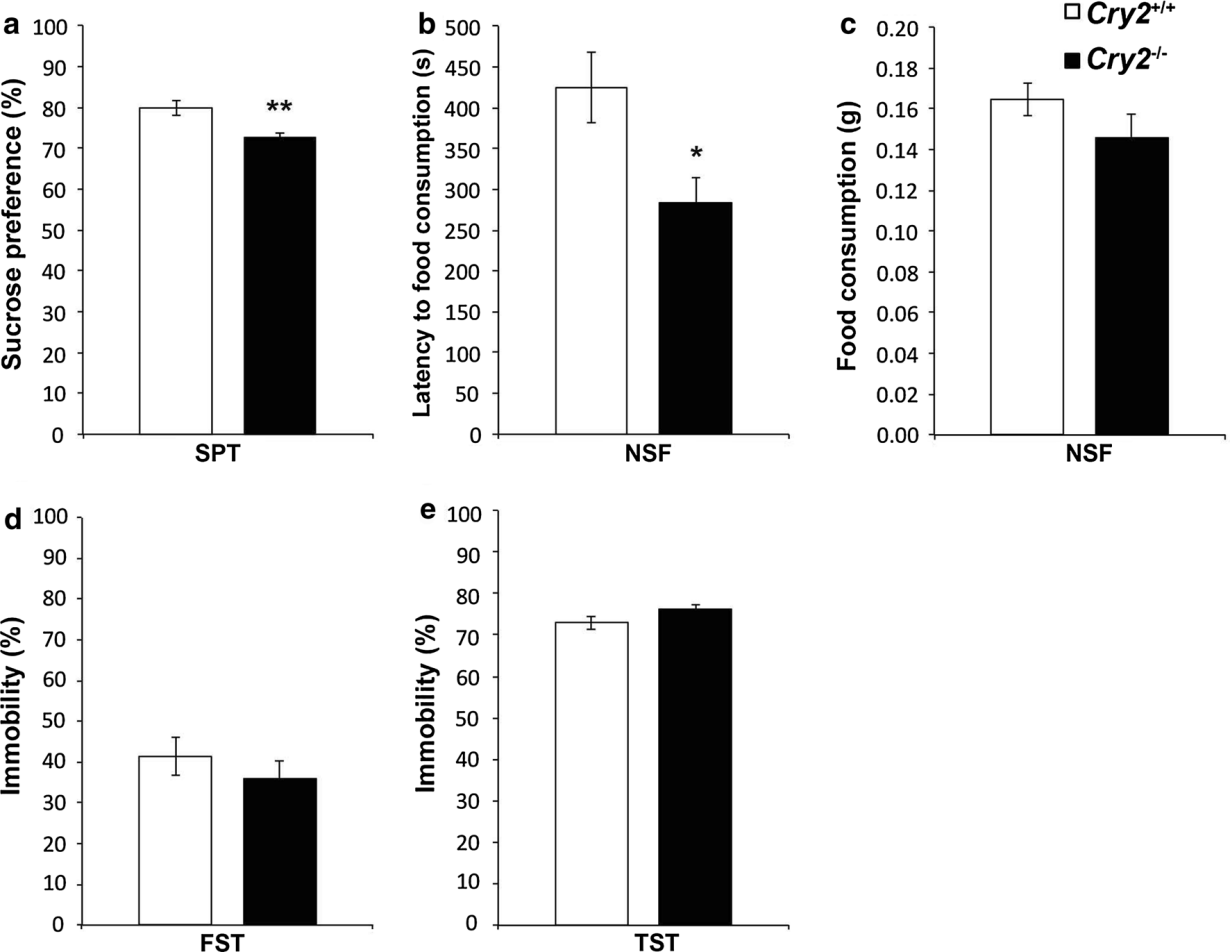
Effect of disruption of the *Cry2* gene on anhedonia, hyponeophagia and behavioral despair. In comparison to wild-type littermates (*Cry2* ^+/+^), *Cry2* ^−/−^ mice displayed **a** lower sucrose preference in the sucrose preference test (SPT) (*n* = 11 per genotype). In the novelty suppressed feeding (NSF) test *Cry2* ^−/−^ mice showed **b** lower latency to food consumption in a novel environment and **c** unaltered food consumption in the home cage (Cry^+/+^ *n* = 11; *Cry2* ^−/−^ *n* = 12). Behavioral despair evaluated in **d** the forced swim test (FST) (*n* = 12 per genotype) and **e** tail suspension test (TST) (Cry^+/+^ *n* = 12; *Cry2* ^−/−^ *n* = 11) was comparable between genotypes. Data are displayed as mean ± SEM. *P < 0.05; **P < 0.01

For the assessment of anhedonic behavior, one of the two core symptoms of depression in humans (DSM-V; American Psychiatric Association 2013), *Cry2* ^−/−^ mice and wild-type littermates *Cry2* ^+/+^ were subjected to the SPT. A significant reduction in the preference for sucrose was found in *Cry2* ^−/−^ mice; *t*_(20)_ = 3.80, *P* < 0.01 (Fig. 1a). No significant differences in body weight were detected between groups [*Cry2* ^+/+^ 25.0 ± 0.3 g, *Cry2* ^−/−^ 24.1 ± 0.6 g; *t*_(22)_ = 1.33, *P* > 0.05].

#### Cry2^−/−^ mice exhibit reduced latency to first feeding event

In order to evaluate depression-related hyponeophagia in *Cry2* ^−/−^ mice, the NSF (Fig. 1b), a standardized test responsive to chronic, but not acute antidepressant treatment (Bodnoff et al. 1989; Dulawa et al. 2004; Merali et al. 2003), was employed. Reduced hyponeophagia, as reflected in a significant decrease in the time to initiate feeding, was observed in *Cry2* ^−/−^ compared to *Cry2* ^+/+^ animals [*t*_(21)_ = 0.51, *P* < 0.05]. No significant differences in home-cage food consumption were detected [*t*_(21)_ = 2.19, *P* > 0.05) (Fig. 1c).

#### Cry2^−/−^ mice demonstrate unaltered behavior in the forced swim test and in the tail suspension test

*Cry2* ^+/+^ and *Cry2* ^−/−^ mice were tested in two standardized paradigms for the assessment of depression-related behavioral despair in rodents, the FST (Fig. 1d) and the TST (Fig. 1e). In both tests, the time spent immobile is used as a parameter indicative of depression-like behavior. No differences between *Cry2* ^+/+^ and *Cry2* ^−/−^ mice were observed in either test.

**Fig. 2:**
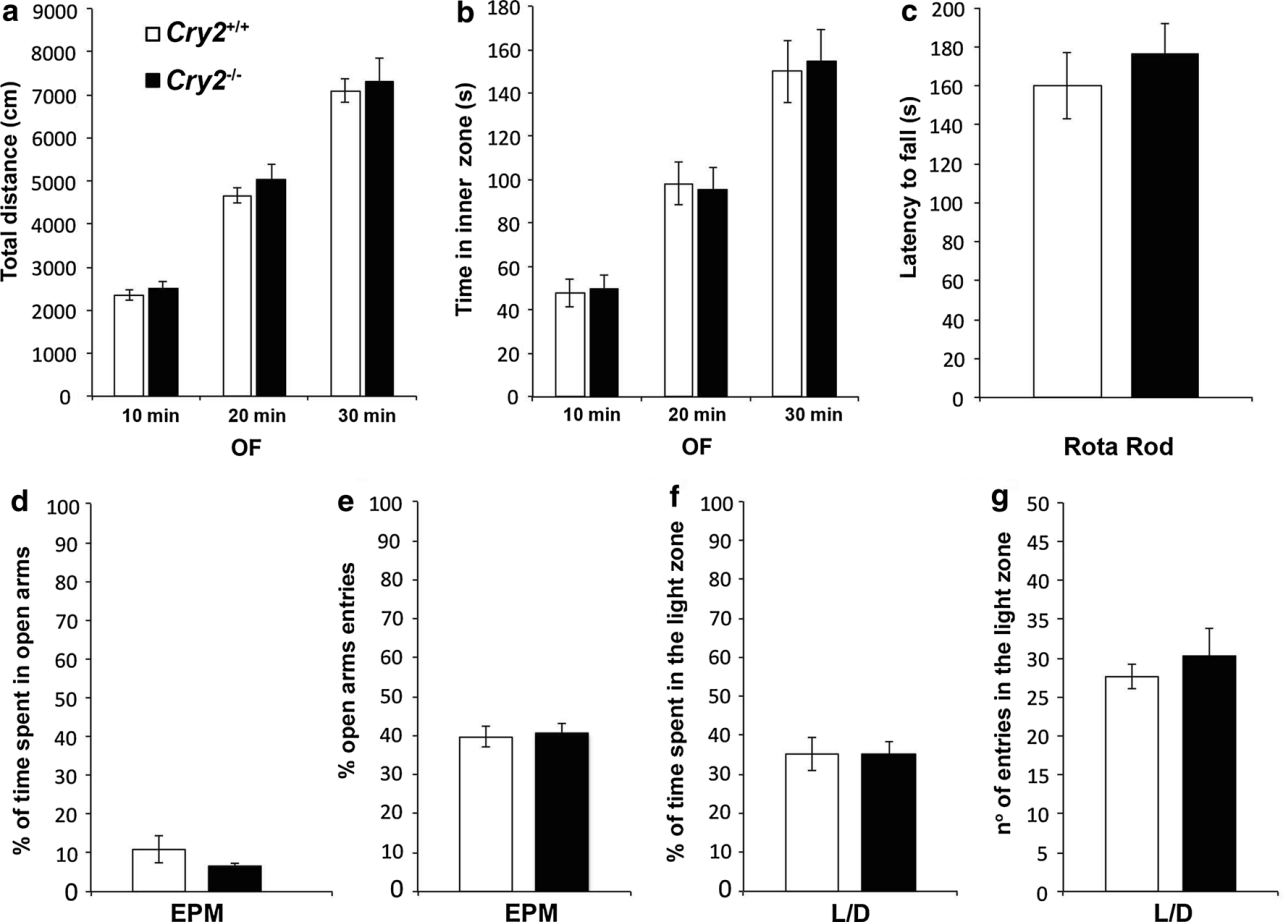
Effect of disruption of the *Cry2* gene on locomotion, motor coordination and anxiety-like behavior. Performance of *Cry2* ^−/−^ in the open field (OF)—**a** total distance traveled and **b** time spent in the center zone—and in the Rota Rod **c** (latency to fall) was comparable to wild-type littermates (*Cry2* ^+/+^). No differences in anxiety-like behavior were observed between genotypes in the elevated plus maze (EPM) in **d** percentage of time spent in open arms and **e** percentage of open arm entries, or in the light/dark box (LD) in **f** percentage of time spent in the light zone and **g** number of entries in the light zone. Data are displayed as mean ± SEM (*n* = 11–12 per genotype)

To control for alterations in locomotor activity or motor coordination potentially confounding the performance in the FST and TST, OF (Fig. 2a, b) analysis and Rota Rod (Fig. 2c) analysis were carried out. No differences between *Cry2* ^−/−^ and *Cry2* ^+/+^ mice were observed in any of the parameters evaluated.

#### Cry2^−/−^ mice showed unaltered anxiety-like behavior

To investigate whether the deficiency in *Cry2* modulated anxiety-like behavior, *Cry2* ^−/−^ and *Cry2* ^+/+^ mice were subjected to the EPM and the LD tests. No differences in anxiety-like behavior, as represented by percentage of time spent in open arms and the percentage of open arms entries in the EPM test (Fig. 2d, e), and time spent and entries into the light zone in the LD test (Fig. 2f, g) were observed between genotypes.

### Diurnal amygdala gene expression profile

**Fig. 3:**
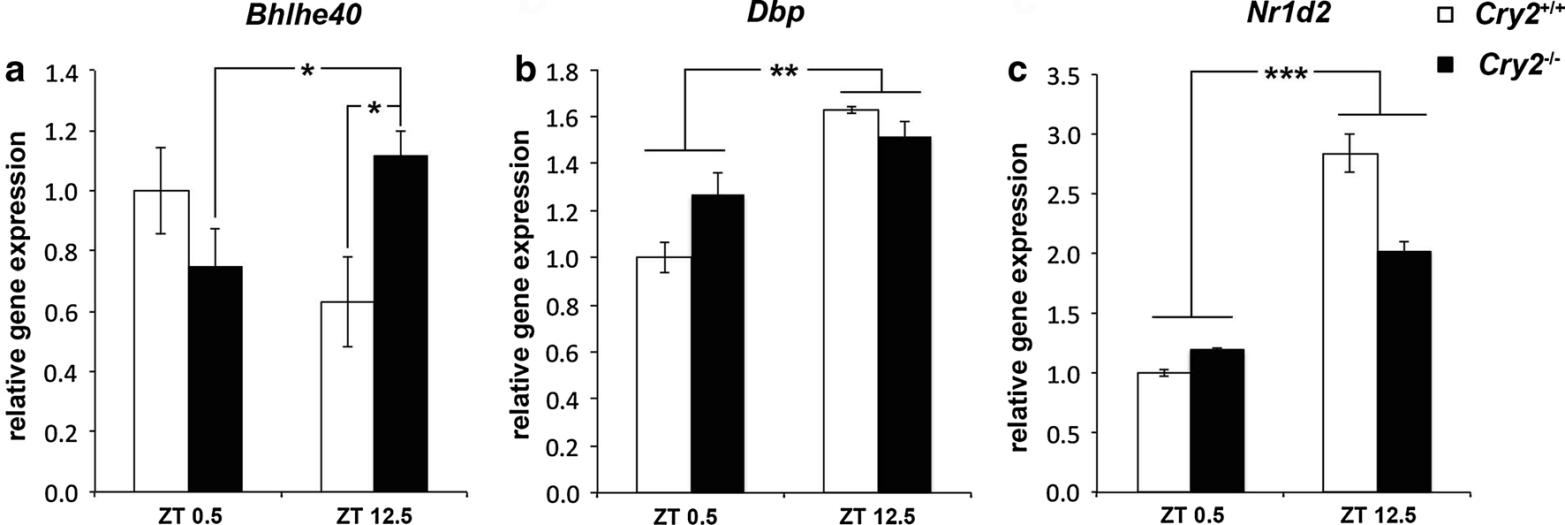
Amygdala clock gene expression in *Cry2* ^−/−^ and *Cry2* ^+/+^ mice at ZT 0.5 and ZT 12.5. Relative gene expression of the clock genes **a** *Bhlhe40*, **b** *Dbp* and **c** *Nr1d2* determined by qRT-PCR in basolateral amygdala tissue of *Cry2* ^+/+^ (*white bars*) and *Cry2* ^−/−^ (*black bars*) mice (*n* = 5–6 per genotype per condition). Results were normalized to β-actin as reference gene and plotted relative to the mean of the control sample at ZT 0.5. Data are displayed as mean ± SEM. *P < 0.05; **P < 0.01; ***P < 0.001

Aiming to explore the molecular correlates of the observed behavioral phenotype of *Cry2* ^−/−^ mice, we focused on gene expression analysis in the basolateral amygdala, critically involved in the regulation of emotional states and related to the pathophysiology of mood disorders (Ressler and Mayberg 2007). Diurnal profiles of clock genes—alterations of which have been previously reported in both depressed patients and animal models of the disease (Li et al. 2013; Savalli et al. 2014)—and neurotrophic and growth factors implicated in the neurobiological alterations of depression were analyzed in *Cry2* ^−/−^ and *Cry2* ^+/+^ mice by qRT-PCR. Mice of both genotypes were randomly divided into two groups and basolateral amygdala samples were collected at two different time points, ZT 0.5 (light) and ZT 12.5 (dark). 20 core clock genes were examined, all of which were found to be expressed in the analyzed samples (Supplementary Table 1a). Two-way ANOVA analysis revealed a statistically significant interaction between genotype and time point [*F*_(1,19)_ = 5.89; *P* < 0.05] for *basic helix*-*loop*-*helix family*, *member e40* (*Bhlhe40*) also known as *deleted in esophageal cancer 1* (*Dec1*) (Fig. 3a), without significant main effect. For *D site of albumin promoter* (*albumin D*-*box*) *binding protein* (*Dbp*), a significant main effect of time point [*F*_(1,19)_ = 9.44; *P* < 0.01; Fig. 3b], without effect of genotype [*F*_(1,19)_ = 0.58; *P* > 0.05] or interaction between genotype and time point [*F*_(1,19)_ = 2.09; *P* > 0.05], was detected. For *nuclear receptor subfamily 1*, *group D*, *member 2* (*Nr1d2*) also known as *Rev*-*erbβ*, a significant main effect of time point [*F*_(1,19)_ = 21.99; *P* < 0.001; Fig. 3c] was observed, but no significant main effect of genotype [*F*_(1,19)_ = 0.24; *P* > 0.05] or interaction between genotype and time point [*F*_(1,19)_ = 2.38; *P* > 0.05] was revealed. No statistically significant differences for the other clock genes analyzed were observed (Supplementary Table 1b).

**Fig. 4:**
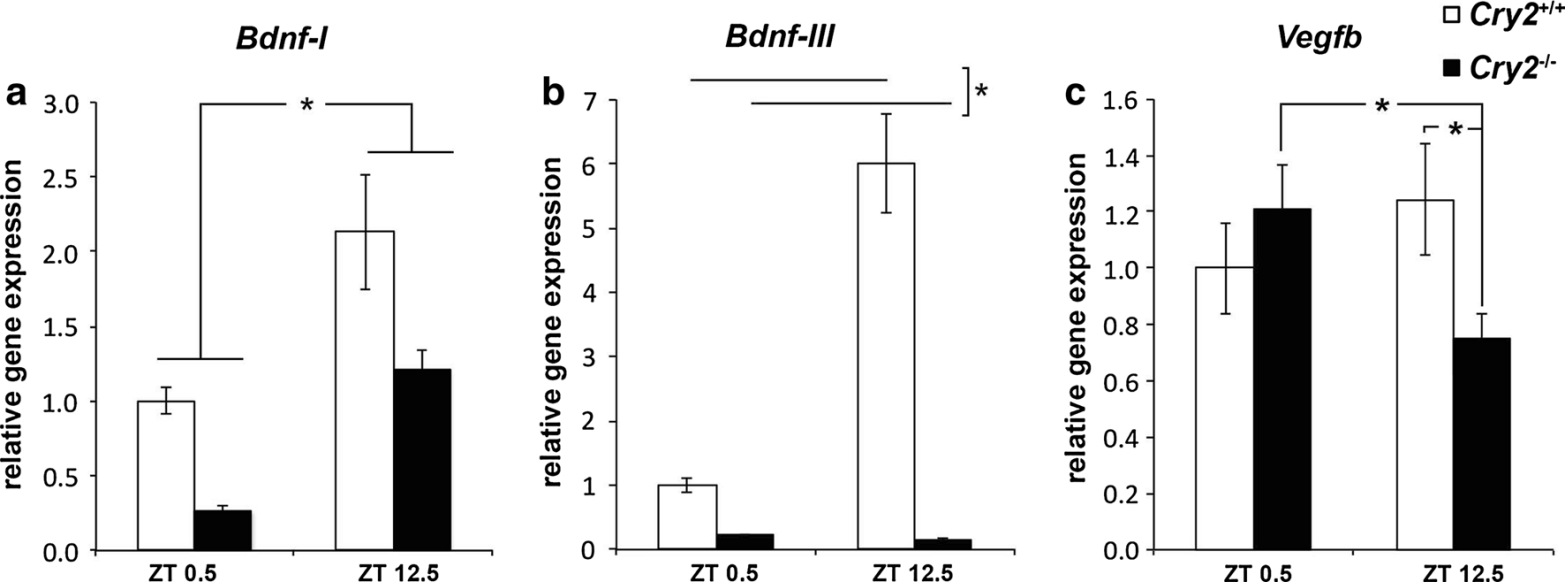
Amygdala neurotrophic factor expression in *Cry2* ^−/−^ mice and *Cry2* ^+/+^ at ZT 0.5 and ZT 12.5. Relative gene expression of **a** *Bdnf*-*I*, **b** *Bdnf*-*III* and **c** *Vegfb* determined by qRT-PCR in basolateral amygdala tissue of *Cry2* ^+/+^ (*white bars*) and *Cry2* ^−/−^ (*black bars*) mice (*n* = 5–6 per genotype per condition). Results were normalized to β-actin, as reference gene and plotted relative to the mean of the control sample at ZT 0.5. Data are displayed as mean ± SEM. *P < 0.05

qRT-PCR analysis of mRNA levels of molecular elements of growth factor systems highly implicated in the pathophysiology of depression (Yu and Chen 2011; Clark-Raymond and Halaris 2013), namely *brain*-*derived neurotrophic factor* (*Bdnf*), *vascular endothelial growth factor* (*Vegf*) and their respective receptors was carried out in basolateral amygdala samples of *Cry2* ^−/−^ and *Cry2* ^+/+^ mice (Supplementary Table 2a). For the individual isoforms of *Bdnf*, two-way ANOVA revealed a significant main effect of time point for *Bdnf*-*I* [*F*_(1,19)_ = 5.61; *P* < 0.05; Fig. 4a]. No significant main effect of genotype [*F*_(1,19)_ = 3.86; *P* > 0.05] or significant genotype by time point interaction [*F*_(1,19)_ = 0.62; *P* > 0.05] was observed. A significant main effect of genotype was observed for *Bdnf*-*III* [*F*_(1,19)_ = 4.91; *P* < 0.05; Fig. 4b]. No significant effect of time point [*F*_(1,19)_ = 0.35; *P* > 0.05] or genotype by time point interaction [*F*_(1,19)_ = 0.91; *P* > 0.05] was revealed.

In the case of members of the *Vegf* family, there was a statistically significant genotype by time point interaction for *Vegfb* [*F*_(1,19)_ = 4.97; *P* < 0.05; Fig. 4c], without significant main effect of either genotype [*F*_(1,19)_ = 1.02; *P* > 0.05] or time point [*F*_(1,19)_ = 0.70; *P* > 0.05]. No statistically significant differences for the other growth factor genes analyzed were observed (Supplementary Table 2b).

## Discussion

The present study firstly assessed the direct consequence of *Cry2*-deficiency on depression-like behavior and examined its molecular signature in the mouse basolateral amygdala. Behaviorally, increased anhedonic behavior of *Cry2* ^−/−^ mice was observed in the SPT and reduced hyponeophagia in the NSF test, without alterations in behavioral despair in the FST or TST. Anxiety-like behavior in the EPM or LD tests was not altered in *Cry2* ^−/−^ mice. Amygdala diurnal gene expression profiling of core clock genes and growth factor systems previously implicated in the pathophysiology of depression revealed that levels of *Bhlhe40* were significantly elevated in the beginning of the active (dark) phase in *Cry2* ^−/−^ mice as compared to wild-type littermates (*Cry2* ^+/+^). Moreover, the genotype had a significant effect in that *Bdnf*-*III* gene expression was blunted in *Cry2* ^−/−^ mice and *Vegfb* levels were significantly reduced in the beginning of the dark phase in *Cry2* ^−/−^ mice.

Considering that anhedonia is one of the core symptoms of depression in humans and that it is commonly used as indicator of depression-like behavior in animal models (Willner et al. 1992), the observed augmented anhedonic behavior in *Cry*2^−/−^ mice supports previous observations that link altered expression of *Cry2* to depression, both in humans (Lavebratt et al. 2010) and in experimental animals (Griesauer et al. 2014; Savalli et al. 2014). Intact performance of *Cry2* ^−/−^ mice in the FST and the TST supports previous findings (De Bundel et al. 2013) and suggests that *Cry2* may be specifically relevant for the anhedonic endophenotype of depression. Hence, as previously suggested (Lim et al. 2012), the neurobiological aberrations related to anhedonia differ from those mediating the display of despair and support the hypothesis that the diversity of clinical features observed in depressed patients may indeed be subserved by distinct underlying molecular mechanisms. Interestingly, while no differences in baseline anxiety-like behavior were detected in the EPM, the LD and the OF, the significantly reduced latency to feed in *Cry2* ^−/−^ mice in the NSF is indicative of reduced hyponeophagia which has been associated with depression-related anxiety. In a different strain of *Cry2* ^−*/*−^ mice, altered performance in the EPM, which was not corroborated by an additional test assessing anxiety-like behavior, has been reported (De Bundel et al. 2013), and differences to our observations in three independent paradigms testing baseline anxiety-like behavior might relate to the specific mouse line used or variations in the individual experimental set-ups.

While seemingly opposing the result in the SPT, the intriguing observation in the NSF, however, suggests that *Cry2* may be involved in or modulate some of the signaling cascades activated by chronic antidepressant drug treatment which is known to induce a decreased latency to feed in the NSF (Dulawa et al. 2004; Mineur et al. 2009; Warner-Schmidt et al. 2011).

The NSF test is unique among the plethora of tests available to evaluate anxiety-like and depression-related behaviors in mice. In comparison to “classical” assays monitoring anxiety-like behavior, such as the EPM and the LD, it comprises the additional aspect of motivation, since through the component to food-deprivation the animal is experiencing a conflict situation in which the innate avoidance for open novel areas has to be reconciled with the incentive to consume food. On the other hand, the SPT also evaluates the ability to seek pleasure from the rewarding experience of energy consumption, which is in the SPT evaluated in the familiar, hence experienced as “safe”, home cage environment. The NSF introduces the elicitation of endogenous fear through the perception of novel, anxiogenic spaces, as added variable. This characteristic set of test-specific demands may explain the distinctive position of the NSF with regard to its potential to selectively reveal behavioral effects of chronic antidepressant treatment. Hence, considering the results of reduced latency to feed in the NSF, it is tempting to speculate that *Cry2* ^−*/*−^ mice might be more sensitive to the effects of antidepressant treatments, despite or even because of their augmented depression-related anhedonia displayed in the NSF. However, this interesting hypothesis warrants further experimental evidence through the assessment of the behavioral and cellular effects of chronic administration of antidepressant drugs in a novel cohort of *Cry2*-deficient mice and wild-type littermates.

Of note, in a mouse model of an anxiety/depression-like state induced by chronic corticosterone treatment, chronic antidepressant treatment has been shown to differentially affect depression-like behavior in specific tests, including the NSF, in a hippocampal neurogenesis-dependent manner. Hence, a particular involvement of *Cry2* in the neurogenesis-dependent molecular pathways stimulated by chronic antidepressant treatment can be speculated to exist (David et al. 2009). Moreover, one remarkable feature of the Cry proteins is that they oppose glucocorticoid receptor activation and that their deficiency doubles the number of dexamethasone-induced genes in primary fibroblasts from *Cry* double-knockout mice (Lamia et al. 2011). A critical balance between Cry proteins is required for proper clock functioning (van der Horst et al. 1999). Of the two Cry proteins, Cry2 has a key role in balancing Cry expression, since it not only acts as a general repressor, but also opposes in specific actions of Cry1, denying Cry1 from accessing to DNA targets too early (Anand et al. 2013). Earlier, altered regulation (down-regulation) of glucocorticoid-responsive genes in the basolateral amygdala (Monje et al. 2011b) and selective deletion of the forebrain type II glucocorticoid receptor (Solomon et al. 2012) have been linked to increased depression-like behavior in mice. *Cry* double-knockout mice display constitutively high levels of circulating corticosterone, suggesting that suppression of the hypothalamic–pituitary–adrenal axis is reduced and glucocorticoid transactivation in the liver is enhanced (Lamia et al. 2011). On the other hand, mice over-expressing the cerebral glucocorticoid receptor are less susceptible to display depression-like behavior (Schulte-Herbruggen et al. 2006). They have constitutively high levels of Bdnf protein in the hippocampus and higher levels of Bdnf protein in the amygdala, especially during the first hour of the passive (light) phase, when corticosteroid levels are low (Schulte-Herbruggen et al. 2006). In agreement, we found herein that *Cry2*-deficiency caused constitutively low levels of *Bdnf*-*III* mRNA in the basolateral amygdala.

It is known that *Bdnf* stimulates *Vegfb* gene expression (Takeda et al. 2005) and that *Vegfb* in turn stimulates neurogenesis in adult mice (Sun et al. 2006). It seems that *Bhlhe40* plays a role in these cascades (Rossner et al. 1997), but it is not known which specific *Bdnf* isoform is modulated. We here found that levels of *Vegfb* mRNA in the basolateral amygdala were reduced in the beginning of the active (dark) phase, suggesting a reciprocal relationship between *Bhlhe40* and *Vegfb* in *Cry2* ^−/−^ mice. Intriguingly, *Bhlhe40*, *Dbp* and *Nr1d2* are among the top-ranked genes that are rhythmically expressed in the human brain, and individuals with major depressive disorder appear to have lost the alignment between *Bhlhe40* and *Per2* expression timetables in six brain regions, e.g. in the amygdala (Li et al. 2013).

Another interesting feature of Cry proteins is that they are the actual repressors of the feedback loops in the core of circadian clocks (Dardente et al. 2007; Ye et al. 2011). In addition to their actions in the cell nucleus, Cry proteins act as inhibitors of adenylyl cyclase, thereby limiting cyclic adenosine monophosphate production (Narasimamurthy et al. 2012) and inhibiting the G protein coupled receptor activity through a direct interaction with the G(s)alpha subunit (Zhang et al. 2010). By these mechanisms of action, as we hypothesize here, Cry proteins might protect the individual from a depression-like state seen in conditions where dysfunction in control of the mesolimbic dopaminergic tracts leads to increased cyclic adenosine monophosphate production and increased depression-like behavior (Park et al. 2005).

However, since our observations are based upon a “conventional” knock-out mouse model, we cannot rule out that compensatory changes induced by *Cry2*-deficiency during development may contribute to the observed behavioral and molecular phenotype of *Cry2* ^−/−^ mice. Moreover, since *Cry2*-deletion in this mouse line is not restricted to the hippocampus, the absence of Cry2 may impact on other physiological activities which could in turn modulate the herein studied brain functions and molecular processes. Furthermore, since here gene expression was evaluated in tissue derived from animals under light-entrained circadian rhythms, the specific relevance of *Cry2* for the behavioral and molecular functions evaluated under free-running conditions needs to be addressed in future studies.

In conclusion, the present study provides evidence that deletion of *Cry2* is associated with depression-like behavior, namely the sucrose preference test, selectively impinging on the anhedonic endophenotype of depression in the mouse. The distinct behavioral display was associated with a modulation of the diurnal pattern of expression of specific clock genes and growth factors in the basolateral amygdala of *Cry2* ^−/−^ mice.

On the basis of these data, we propose that *Cry2* exerts a role in the control of emotional states by participating in amygdala functioning, acting on molecular elements of both the endogenous clock machinery and the network of neurotrophic support.

## Electronic supplementary material

Below is the link to the electronic supplementary material.

Supplementary material 1 (DOCX 16 kb)

Supplementary material 2 (XLSX 10 kb)

Supplementary material 3 (XLSX 10 kb)

Supplementary material 4 (XLSX 10 kb)

Supplementary material 5 (XLSX 9 kb)
