# Supplementary material for: Anhedonic behavior in cryptochrome 2-deficient mice is paralleled by altered diurnal patterns of amygdala gene expression
Source: Amino Acids. 2015 Mar 28;47(7):1367–77. doi: 10.1007/s00726-015-1968-3 (PMC4458264; doi:10.1007/s00726-015-1968-3)
Supplement: Supplementary file 1 — Supplementary material 1 (DOCX 16 kb) [file 726_2015_1968_MOESM1_ESM.docx]

**Supplementary Materials S1**

The sequences of clock genes primers were as follows:

*Clock*, forward, 5’-GGCGTTGTTGATTGGACTAGG-3’, reverse, 5’-GAATGGAGTCTCCAACACCCA-3’;

*Bmal1*, forward, 5’-AACCTTCCCGCAGCTAACAG-3’; reverse, 5’-AGTCCTCTTTGGGCCACCTT-3’;

*Cry1*, forward, 5’-AGGAGGACAGATCCCAATGGA-3’; reverse, 5’-GCAACCTTCTGGATGCCTTCT-3’;

*Cry2*, forward, 5’-AGCTGATGTGTTCCCAAGGCT-3’; reverse, 5’-CATAATGGCTGCATCCCGTT-3’;

*CycloB*, forward, 5’-GGTGGAGAGCACCAAGACAGA-3’; reverse, 5’-GCCGGAGTCGACAATGATG-3’;

*Dbp*, forward, 5’-GGAACTGAAGCCTCAACCAATC-3’; reverse, 5’-CTCCGGCTCCAGTACTTCTCA-3’;

*E4bp4*, forward, 5’-AGAACCACGATAACCCATGAAAG-3’; reverse, 5’-GACTTCAGCCTCTCATCCATCAA-3’;

*Id2*, forward 5’-AGGCATCTGAATTCCCTTCTGA-3’; reverse, 5’-AGTCCCCAAATGCCATTTATTTAG-3’;

*Npas2*, forward, 5’-ACGCAGATGTTCGAGTGGAAA-3’; reverse, 5’-CGCCCATGTCAAGTGCATT-3’;

*Per1*, forward, 5’-CCAGATTGGTGGAGGTTACTGAGT-3’; reverse, 5’-GCGAGAGTCTTCTTGGAGCAGTAG-3’;

*Per2*, forward, 5’-AGAACGCGGATATGTTTGCTG-3’; reverse, 5’-ATCTAAGCCGCTGCACACACT-3’;

*Per3*, forward, 5’-CCGCCCCTACAGTCAGAAAG-3’; reverse, 5’-GCCCCACGTGCTTAAATCCT-3’;

*Nr1d1 (Rev-erbα)*, forward, 5’-CCCTGGACTCCAATAACAACACA-3’; reverse, 5’-GCCATTGGAGCTGTCACTGTAG-3’;

*Nr1d2 (Rev-erbβ)*, forward, 5’-GGAACGGACCGTCACCTTT-3’; reverse, 5’-TCCCCTGCTCCCATTGAGT-3’;

*Bhlhe40 (Dec1)*, forward, 5’-CCCGTCTCTGATGAATAAAGACCA-3’; reverse, 5’-GGACAGCATGCCGTAGAAGTGA-3’;

*Bhlhe41 (Dec2)*, forward, 5’-ATGAATGCATTGCTCAGCTGAAAG-3’; reverse, 5’-GCTGCTGCTCAGTTAAGGCTGTTAG-3’;

*ROR-α*, forward, 5’-TTGCCAAACGCATTGATGG-3’; reverse, 5’-TTCTGAGAGTCAAAGGCACGG-3’;

*ROR-β*, forward, 5’-ATGGCAGACCCACACCTACG-3’; reverse, 5’-TATCCGCTTGGCGAACTCC-3’;

*ROR-γ*, forward, 5’-CGAGATGCTGTCAAGTTTGGC-3’; reverse, 5’-TGTAAGTGTGTCTGCTCCGCG-3’;

*NeuroD1*, forward, 5’-CGAGTCATGAGTGCCCAGCTTA-3’; reverse, 5’-CCGGGAATAGTGAAACTGACGTG-3’.
